# Anti-Müllerian hormone (AMH) autocrine signaling promotes survival and proliferation of ovarian cancer cells

**DOI:** 10.1038/s41598-021-81819-y

**Published:** 2021-01-26

**Authors:** Maëva Chauvin, Véronique Garambois, Pierre-Emmanuel Colombo, Myriam Chentouf, Laurent Gros, Jean-Paul Brouillet, Bruno Robert, Marta Jarlier, Karen Dumas, Pierre Martineau, Isabelle Navarro-Teulon, David Pépin, Thierry Chardès, André Pèlegrin

**Affiliations:** 1 IRCM, Institut de Recherche en Cancérologie de Montpellier, Campus Val d’Aurelle, 34298 Montpellier Cedex, France; 2 INSERM, U1194, 34298 Montpellier, France; 3 Université de Montpellier, 34298 Montpellier, France; 4 Institut Régional du Cancer de Montpellier, ICM, 34298 Montpellier, France; 5 Département de Biochimie et Biologie Moléculaire, CHU de Nîmes, Nîmes, France; 6 SurgiMAb, 10 Parc Club du Millénaire, 1025 Avenue Henri Becquerel, 34000 Montpellier, France; 7 Department of Surgery, Harvard Medical School, Boston, MA USA; 8 Pediatric Surgical Research Laboratories, Massachusetts General Hospital, Boston, MA USA

**Keywords:** Cancer therapy, Gynaecological cancer, Cell signalling

## Abstract

In ovarian carcinoma, anti-Müllerian hormone (AMH) type II receptor (AMHRII) and the AMH/AMHRII signaling pathway are potential therapeutic targets. Here, AMH dose-dependent effect on signaling and proliferation was analyzed in four ovarian cancer cell lines, including sex cord stromal/granulosa cell tumors and high grade serous adenocarcinomas (COV434-AMHRII, SKOV3-AMHRII, OVCAR8 and KGN). As previously shown, incubation with exogenous AMH at concentrations above the physiological range (12.5–25 nM) decreased cell viability. Conversely, physiological concentrations of endogenous AMH improved cancer cell viability. Partial AMH depletion by siRNAs was sufficient to reduce cell viability in all four cell lines, by 20% (OVCAR8 cells) to 40% (COV434-AMHRII cells). In the presence of AMH concentrations within the physiological range (5 to 15 pM), the newly developed anti-AMH B10 antibody decreased by 25% (OVCAR8) to 50% (KGN) cell viability at concentrations ranging between 3 and 333 nM. At 70 nM, B10 reduced clonogenic survival by 57.5%, 57.1%, 64.7% and 37.5% in COV434-AMHRII, SKOV3-AMHRII, OVCAR8 and KGN cells, respectively. In the four cell lines, B10 reduced AKT phosphorylation, and increased PARP and caspase 3 cleavage. These results were confirmed in ovarian cancer cells isolated from patients’ ascites, demonstrating the translational potential of these results. Furthermore, B10 reduced COV434-MISRII tumor growth in vivo and significantly enhanced the median survival time compared with vehicle (69 *vs* 60 days; p = 0.0173). Our data provide evidence for a novel pro-survival autocrine role of AMH in the context of ovarian cancer, which was targeted therapeutically using an anti-AMH antibody to successfully repress tumor growth.

## Introduction

Anti-Müllerian hormone (AMH) is a member of the TGFβ family, and acts by binding to its specific receptor (AMH type II receptor; AMHRII) that recruits type I receptors (AMHRI: ALK2, ALK3 and ALK6). AMHRI phosphorylation induces SMAD 1/5/8 phosphorylation and their migration into the nucleus where through SMAD4, they regulate different responsive genes, depending on the target tissue^1^. AMH was first described as the hormone responsible for Müllerian duct regression during male development^2^, and therefore, it is almost only considered as an anti-proliferative hormone. However in the TGFβ family, background matters^3^, and during male development, Müllerian duct regression requires also epithelial-mesenchymal transition (EMT) and is finely tuned by AMHRII expression^4^. More globally, AMH can be considered as a gonadal cytokine^5^.

AMH has been proposed as a potential treatment for gynecologic tumors since 1979^6^, based on the observation by RE Scully that epithelial ovarian carcinoma resembles histologically the tissues derived from Müllerian ducts^7^. Currently, it is known that the induction of Müllerian duct regression by AMH involves different cell types and mechanisms^8^, but its use as a potential apoptosis inducer against cancer cells is now well documented (reviewed in^9^). Indeed, in AMHRII-positive cancer cells, treatment with high concentrations of exogenous AMH inhibits proliferation and induces apoptosis^10^. However, the use of recombinant AMH has been hampered by the difficulties linked to the production of sufficient amounts of bioactive AMH and to its delivery at the tumor site^11^. Production and purification of recombinant AMH is still a challenge^12^, although Pépin et al. recently described an original production strategy and an alternative delivery approach using gene therapy to be translated in clinical phase^13,14^.

To overcome this challenge and to determine whether lower doses of AMH could be used, we wanted to identify the lowest AMH concentration that can induce apoptosis and tumor regression. To our surprise, we found that in addition to the well-known effect on apoptosis induction at high concentration, AMH can induce cancer cell proliferation at lower concentrations. We observed this effect in four ovarian cancer cell lines (derived from two epithelial ovarian tumors and from two sex cord-stromal tumors, including one granulosa cell tumor). Specifically, at physiological concentrations (10 pM), AMH promoted cancer cell viability through ALK2 recruitment. Conversely, in the presence of supraphysiological concentrations of exogenous AMH (25 nM), ALK3 is recruited, heterodimerizes with AMHRII, and drives the apoptotic effects (Chauvin et al. submitted). The effect on proliferation/viability was inhibited by anti-AMH siRNAs and by the new anti-AMH monoclonal antibody B10 characterized in this study. This antibody reduced cell viability and increased apoptosis in four ovarian cancer cell lines and in ascites cells from patients with ovarian cancer. Moreover, the B10 antibody decreased tumor growth in vivo in an ovarian mouse preclinical model. Altogether, our data provide a new vision of AMH in the context of ovarian cancer and open the way to an innovative therapeutic approach to suppress AMH proliferative effect.

## Results

### Cell survival promoted by physiological AMH concentration is abrogated by siRNA-mediated AMH silencing

**Figure 1:**
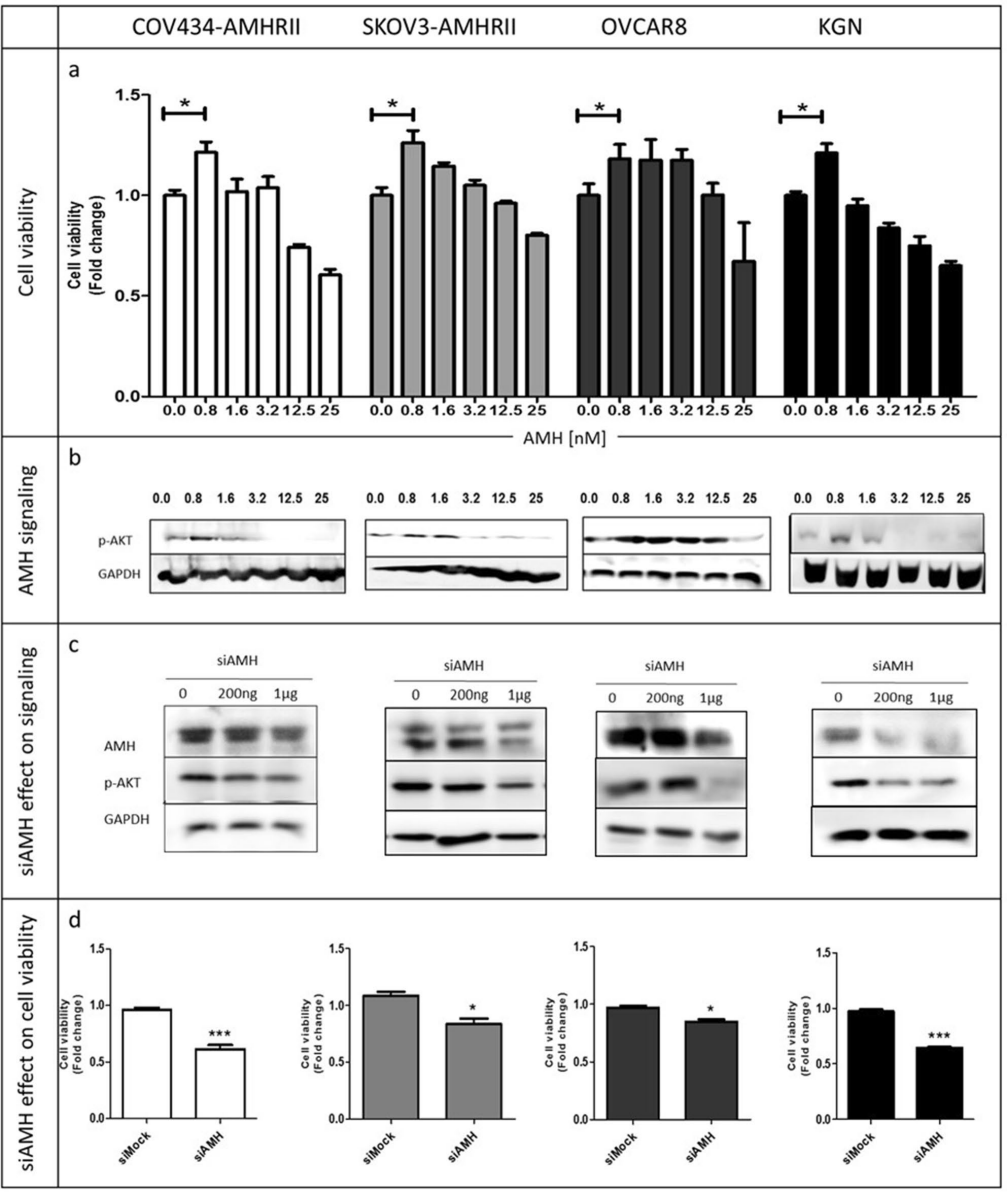
Low-dose recombinant AMH (LR-AMH) promotes cell viability in COV434-AMHRII, SKOV3-AMHRII, OVCAR8 and KGN cells. (**a**) Cell viability (MTS assay) and (**b**) AKT phosphorylation (western blotting) after incubation with 0.8 to 25 nM LR-AMH for 3 days. (**c**) AMH expression at 48 h after transfection of siRNAs against AMH, and (**d**) Effect of siRNA-mediated AMH silencing on cell viability at day 3 post-transfection. Figure shows a representative experiment out of 2 to 4 experiments performed depending on the cell line (mean ± SEM of 3 replicate wells) *p < 0.05; ***p < 0.001. For western blot figure preparation, all proteins were analyzed on the same gel and membrane. After blotting, the lines corresponding to the different proteins were cut and may have been exposed for different lengths of time because the objective was not to compare the different proteins with each other, but to see the effect of AMH (**b**) or of siRNA (**c**) on the expression of each protein.

First, we analyzed the effect of AMH concentration on cell survival in four AMHRII-positive ovarian cancer cell lines, COV434-AMHRII (sex cord stromal tumor), SKOV3-AMHRII (high grade serous adenocarcinoma), OVCAR8 (high grade serous adenocarcinoma), and KGN (granulosa cell tumor), using an MTS assay to measure cell viability and proliferation. In all cell lines, cell viability was significantly increased by the lowest tested concentrations of active recombinant human AMH (0.8 nM LR-AMH), whereas it was reduced by incubation with LR-AMH doses above the physiological range (Fig. 1a). To analyze the involvement of AMH non-canonical signaling pathways^15,16^, we monitored AKT phosphorylation and found that it was decreased upon incubation with LR-AMH, as observed for cell viability (Fig. 1b).

We then transfected the four cell lines with siRNAs against AMH. Due to the important AMH production by these cell lines, particularly in COV434-AMHRII cells, and despite the use of a pool of endoribonuclease-prepared siRNAs, we could not fully silence AMH (western blotting in Fig. 1c). However, this partial AMH depletion was sufficient to reduce AKT phosphorylation (Fig. 1c) and cell viability in all four cell lines, by 20% (OVCAR8 cells) to 40% (COV434-AMHRII cells) (Fig. 1d). Altogether, these results showed that low exogenous AMH concentration promotes cell survival in ovarian cancer cells, and induces AKT phosphorylation, a pro-survival signal.

### The anti-AMH antibody B10 inhibits proliferation of ovarian cancer cells

To test whether an antibody could block the pro-survival mechanisms induced by physiological endogenous AMH as a potential therapeutic strategy, we produced a new monoclonal antibody against AMH.

We isolated the B10 antibody from the human scFv phage display library Husc I^17,18^ after panning using the Ala453-Arg560 AMH cTER domain that is bioactive despite its lower activity compared with cleaved AMH prodomain-mature heterodimers^19,20^. First, we characterized B10 affinity for AMH by ELISA (EC_50_ = 50.4 ± 1.2 nM), and its capacity to inhibit the apoptotic effect of exogenous LR-AMH at high concentrations (25 nM) in COV434-AMHRII and SKOV3-AMHRII cells (Fig. S1). Caspase-3/7 activity induced by 25 nM LR-AMH (fold change relative to untreated cells) was reduced by about 40% in the presence of about 66 nM of B10 (Fig. S1).

**Figure 2:**
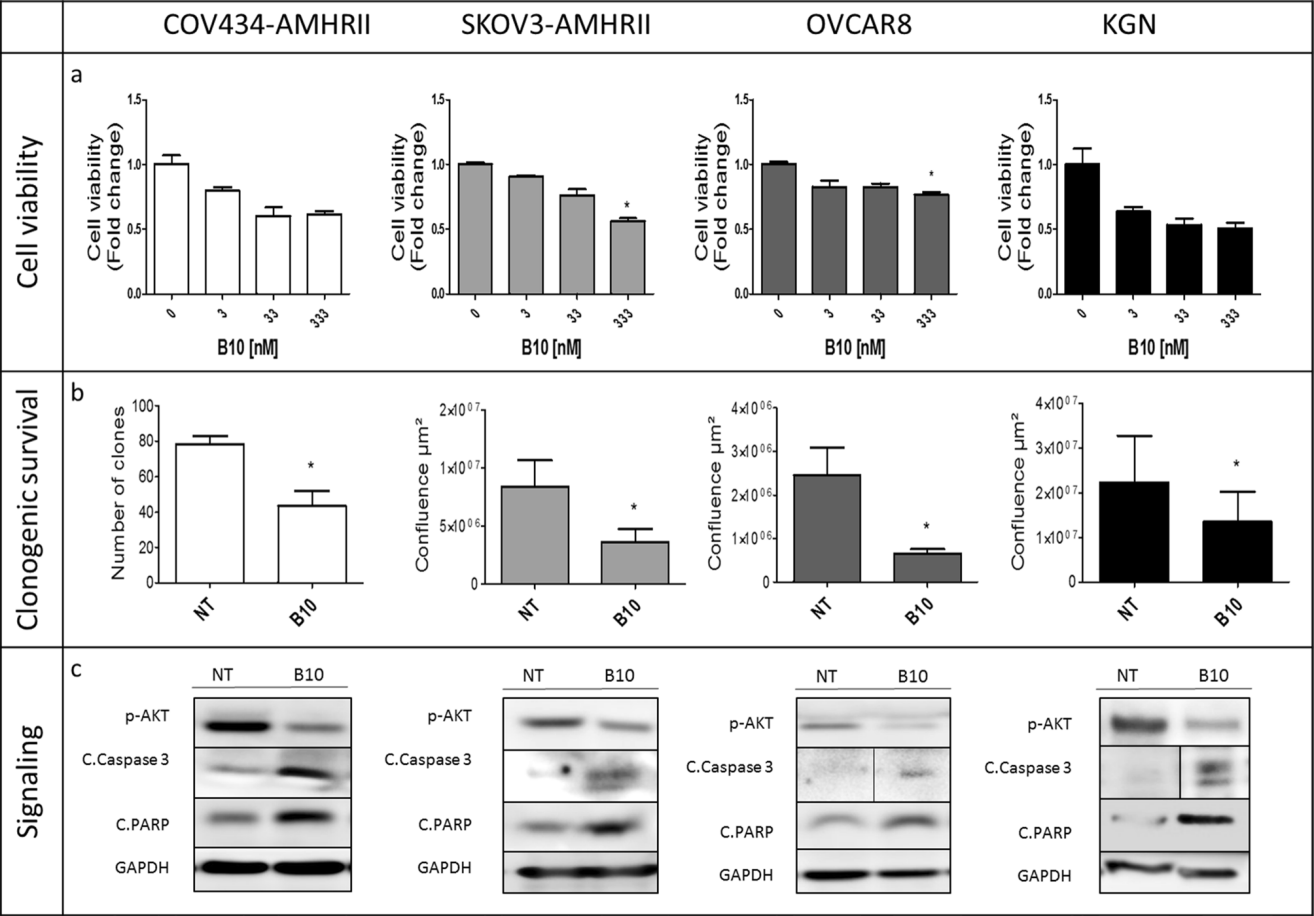
The anti-AMH antibody B10 reduces cell viability/proliferation and induces growth inhibition in COV434-AMHRII, SKOV3-AMHRII, OVCAR8, and KGN cells. (**a**) Cell viability was analyzed (MTS assay) after incubation with 3 to 333 nM B10 for 3 days. (**b**) Clonogenic survival in COV434-AMHRII cells (direct clone counting) and in SKOV3-AMHRII, OVCAR8, and KGN cells (measurement of cell confluence using the Celigo Imaging System) after incubation or not with 70 nM B10 for 11 days. (**c**) AKT phosphorylation and apoptosis induction (cleaved caspase 3 and PARP) after incubation or not with 333 nM B10 for 24 h. All proteins were analyzed on the same gel and membrane. After blotting, the lines corresponding to the different proteins were cut and may have been exposed for different lengths of time because the objective was not to compare the different proteins with each other, but to see the effect of B10 on the expression of each protein. Figure shows a representative experiment out of 2 to 4 experiments performed depending on the cell line (mean ± SEM of 3 replicate wells) *p < 0.05.

We then assessed B10 effect on cell viability in the presence of concentrations of endogenous AMH within the physiological range (5 to 15 pM in culture, as determined in Fig. S2). Depending on the cell line, incubation with B10 induced a decrease by 25% (OVCAR8) to 50% (KGN) of cell viability at antibody concentrations ranging between 3 and 333 nM (Fig. 2a). Moreover, 70 nM of B10 significantly reduced clonogenic survival by 57.5%, 57.1%, 64.7% and 37.5% in COV434-AMHRII, SKOV3-AMHRII, OVCAR8 and KGN cells, respectively (Fig. 2b). In the four cell lines, B10 reduced AKT phosphorylation, and increased PARP and caspase 3 cleavage (Fig. 2c).

**Figure 3:**
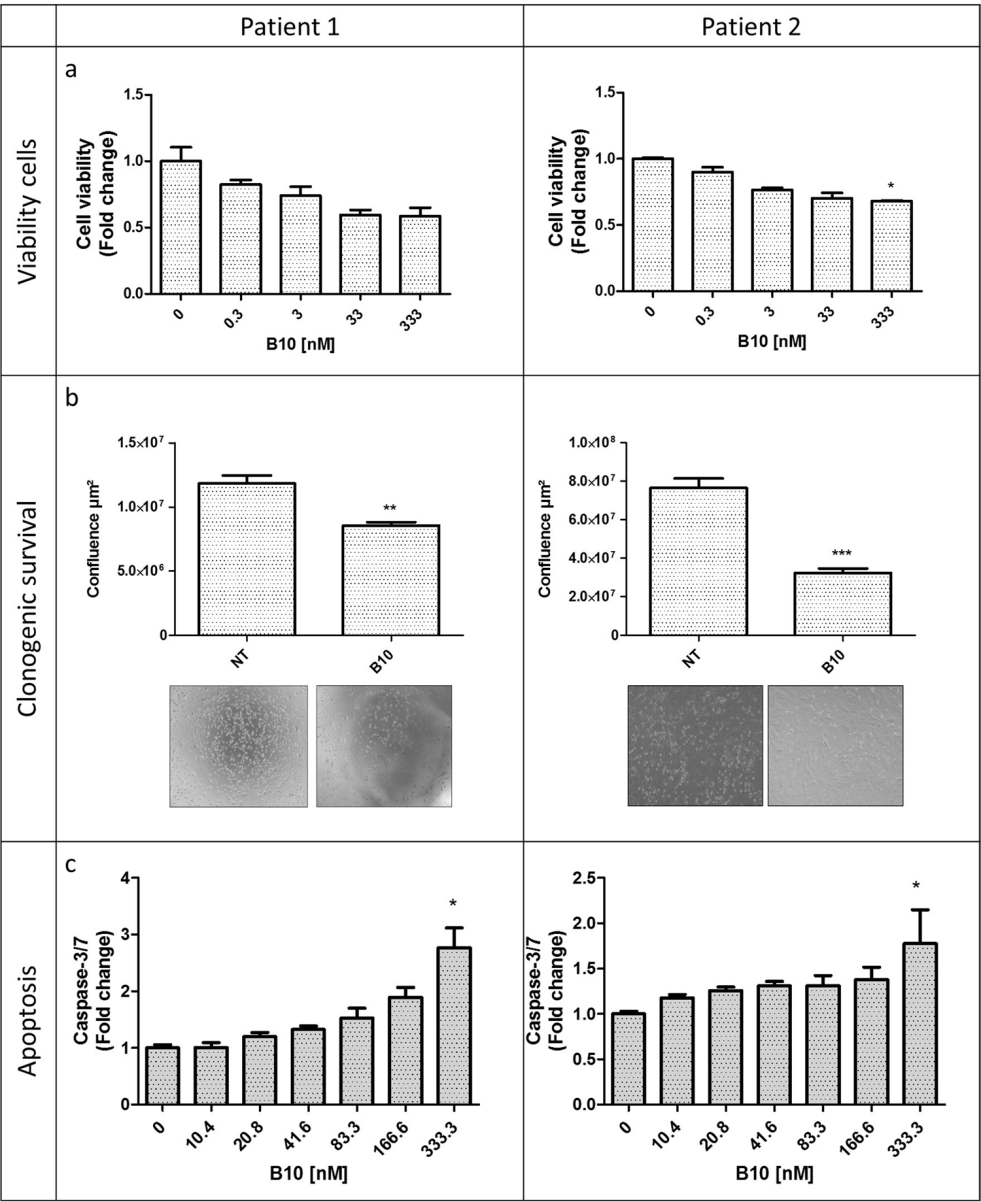
The anti-AMH antibody B10 reduces cell viability/proliferation and induces growth inhibition in tumor cells from ovarian carcinoma ascites samples. (**a**) Cell viability analyzed with the MTS assay after incubation with 0.3 to 333 nM B10 for 3 days. (**b**) Cell growth inhibition (cell confluence measured with the Celigo Imaging System) after incubation or not (NT) with 70 nM B10 for 48 h. (**c**) Apoptosis induction (caspase 3/7 activity) after incubation or not with increasing concentrations of B10. Figure shows mean ± SEM of 3 replicate wells. *p < 0.05; **p < 0.01; ***p < 0.001.

Finally, we assessed the effect of B10 in primary cancer cells isolated from ascites samples of two patients with high grade serous ovarian cancer. These patients were awaiting surgical intervention and had never received chemotherapy. Like in the four cell lines, B10 reduced cell viability by 30% and 20% (patient 1 and 2, respectively) (Fig. 3a), and inhibited cell growth (estimated by the confluence area) by 25% and 65% (patient 1 and 2, respectively) (Fig. 3b), while it increased caspase-3/7 activity up to 3 times (Fig. 3c). Despite the limited number of samples, these results highlight the potential translational application of blocking AMH proliferative effect with specific antibodies.

### The anti-AMH antibody B10 reduces COV434-MISRII tumor growth in vivo

**Figure 4:**
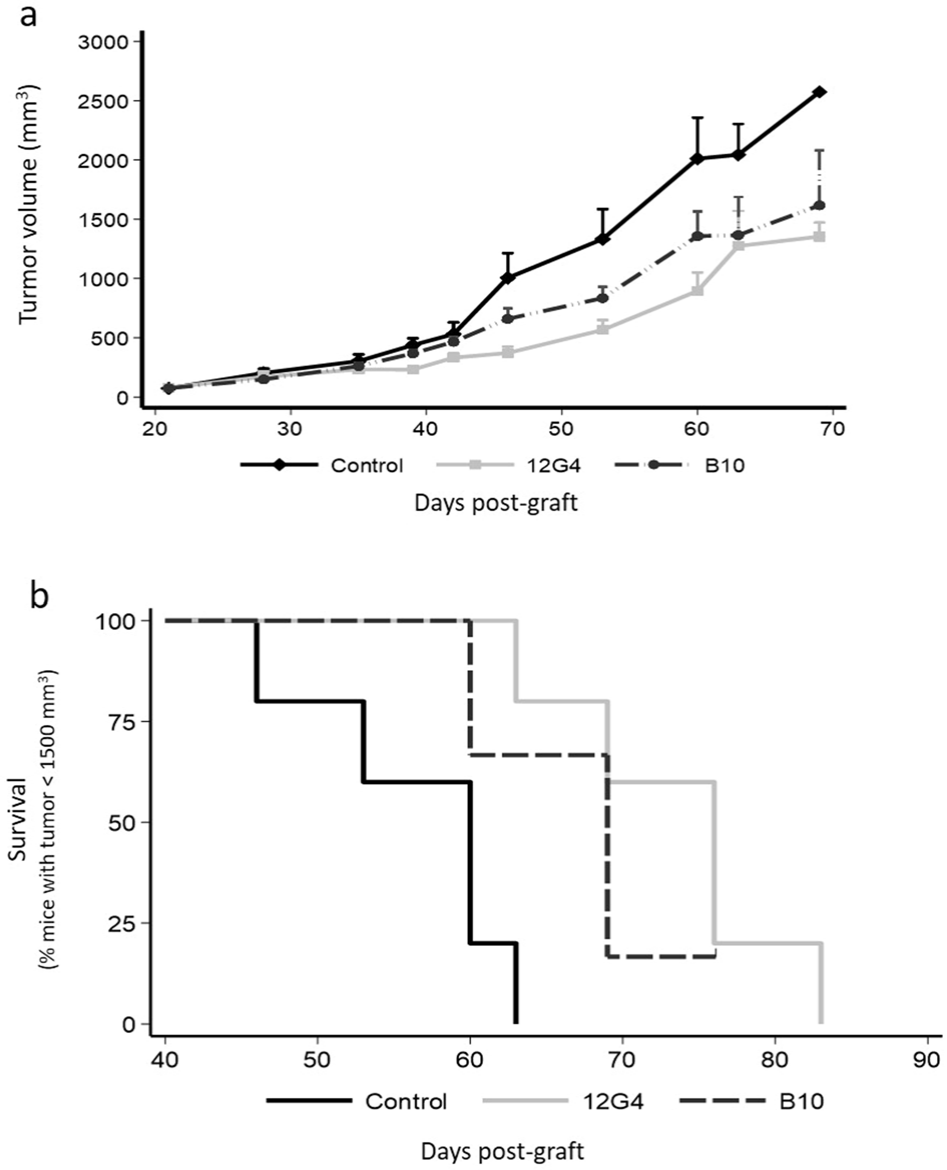
The anti-AMH antibody B10 reduces COV434-MISRII tumor growth in vivo*.* Nude mice bearing COV434-MISRII cell tumors were treated with B10 (anti-AMH antibody), 12G4 (anti-AMHRII antibody) (10 mg/kg/injection for both), or vehicle (NaCl; control) twice a week for 4 weeks. (**a**) Tumor growth curves (mean + 95% confidence intervals), and (**b**) Kaplan–Meier survival curves (percentage of mice with a tumor volume < 1500 mm^3^ as a function of time after graft).

To evaluate whether B10 anti-proliferative effect in vitro could translate into an anti-tumor activity in vivo, we treated mice harboring established COV434-MISRII cell-derived tumors (5 to 7 mice/group) with B10 (anti-AMH antibody), 12G4 (anti-AMHRII antibody) (10 mg/kg/injection for both), or vehicle (NaCl) by intraperitoneal injection twice per week for 4 weeks. Both B10 and 12G4 inhibited tumor growth compared with vehicle (p < 0.001) (Fig. 4a). The median survival times, defined as the time when 50% of mice had a tumor of 1500 mm^3^, were 60, 69 and 76 days for mice treated with vehicle, B10 and 12G4, respectively (p = 0.0050 and p = 0.0173 for 12G4 and B10 vs control; p = 0.4331 between 12G4 and B10) (Fig. 4b).

## Discussion

Many studies, reviewed by Kim JH et al., validated the potential application of AMH as a bio-drug^9^ for ovarian cancer^10,21–24^, cervical and endometrial cancer^25,26^ as well as non-Müllerian tumors, such as breast^27^ and prostate cancer^28^. These studies showed that at experimental doses above the physiological range, exogenous AMH inhibits cancer cell growth in vitro and in vivo, in cell lines and in patient samples. Interestingly, recent results suggest that AMH could be efficient also in chemotherapy-resistant cancer cells and cancer stem cells^29^. However, the major issue for the clinical application of this strategy is the availability of high amounts of clinical-grade bioactive AMH. To address this need, modifications were introduced in the protein (i.e. LR-AMH with an albumin leader sequence and a cleavage site modification) that allow the production of highly potent recombinant AMH^13^. In the present study, we used LR-AMH and confirmed its activity and potential usefulness in preclinical studies. We also compared it with the opposite strategy in which endogenous AMH is neutralized using a specific monoclonal antibody.

The common point of these previous studies is that they all used exogenous AMH at concentration far above the physiological range, typically from 25 to 200 nM, to treat cancer cells. These concentrations are higher than the highest AMH serum concentration observed physiologically (boys from birth to puberty), which are roughly in the 0.3 to 3 nM range^30^, and even higher than the highest intrafollicular concentration measured in women (maximum of 1000 ng/ml, i.e. around 15 nM^31^). This is perfectly logical because this strategy is based on apoptosis induction by AMH during Müllerian duct regression. We obtained similar results in the present study, but we also observed that at more physiological concentrations, AMH promoted cell survival/proliferation in ovarian cancer cells (Fig. 1a). Such paradoxical effect was described by Rehman et al. in mouse Sertoli cells^32^. Indeed, they found that incubation with high concentrations of AMH (0.71 to 11.4 nM, 50 to 800 ng/ml) induces apoptosis, whereas the lowest concentration (0.14 nM, 10 ng/ml) promotes Sertoli cell proliferation. This effect was correlated with increased *PCNA* mRNA levels and ERK phosphorylation. In this physiological situation, AMH could play the dual role needed for Sertoli cell development. Moreover, Beck et al. showed that in lung cancer, AMH/AMHRII signaling regulates EMT and promotes cell survival/proliferation^15^. They suggested that AMH/AMHRII signaling in EMT regulation was important for chemoresistance. Wang PY et al. demonstrated that AMH can act as a motor neuron survival factor in vitro^33^. Based on the expression of AMH and its receptors by motor neurons, they suggested that AMH could be an autocrine factor for these cells^33^. AMH-induced cell survival was also evaluated by Yin et al. in sperm^34^. They suggested that AMH could induce cell survival through a new receptor, YWK-II, in AMHRII-negative cells^34^.

In the present study using anti-AMH siRNAs, we identified the involvement of physiological concentrations of endogenous AMH in the survival of AMHRII-positive ovarian carcinoma cells (Fig. 1d). We then showed that the new anti-AMH antibody B10 can reduce cell viability, clonogenic survival, and AKT phosphorylation in four ovarian cancer cell lines (Fig. 2) and also in tumor cells isolated from ovarian cancer ascites samples (Fig. 3). Interestingly, the B10-induced increase of PARP and caspase 3 cleavage (Fig. 2c) mirrors the effect observed at exogenous LR-AMH concentrations above the physiological range^9^ (and Chauvin et al. submitted). All these in vitro data indicate that inhibition of endogenous AMH with an antibody can mimic the effect of exposure to exogenous AMH concentrations above the physiological range. As the first step towards the in vivo proof of this concept, we showed that in mice, the B10 anti-AMH antibody reduced the growth of COV434-AMHRII cell-derived tumors and significantly increased their median survival time compared with the control group (no treatment) (Fig. 4).

An important factor in evaluating B10 translational potential is AMH intra-tumoral concentration. AMH serum concentration is used for the follow-up of granulosa tumors^35^, but it is below the detection limits in other ovarian cancers. Conversely, to our knowledge, there is no information on AMH concentration in tumors. Recently, Gowkielewic et al. analyzed AMH expression in 232 endometrial tumors by immunohistochemistry, and found that it was expressed in 23 samples^36^. However, the authors did not analyze AMH potential role in tumor development^36^.

Based on our results, we propose that anti-AMH antibodies, such as B10, could represent an innovative therapeutic approach to suppress AMH proliferative effect. Anti-ligand antibodies (e.g. bevacizumab^37^) have been used in oncology for many years, and also for other indications (e.g. anti-TNF antibodies for Crohn’s disease and rheumatoid arthritis^38^). An AMH trapping strategy could be first evaluated in gynecological tumors where the AMH/AMHRII signaling pathway is well described, or in colorectal cancer in which the AMH gene is upregulated^39^ and high AMH RNA expression is an unfavorable prognostic factor (n = 597 patients with a follow-up of more than 12 years; https://www.proteinatlas.org/ENSG00000104899-AMH/pathology).

## Materials and methods

### AMH production and assay

Active recombinant AMH (LR-AMH^13,14^) was produced in CHO cells (Evitria AG, Zürich, Switzerland) according to the WO2014/164891 patent. LR-AMH is a full-length protein and completely cleaved, thus combining efficiency and stability^13,19^. It contains (i) the 24AA leader sequence of albumin instead of the AMH leader sequence to increase production and secretion, and (ii) the RARR/S furin/kex2 consensus site instead of the native AMH RAQR/S sequence at position 423–428 to improve cleavage. AMH was quantified using the Elecsys AMH PLUS kit (Roche Diagnostics). All experiments involving LR-AMH were performed in culture medium containing 1% fetal bovine serum (FBS) to minimize the contribution of bovine AMH that can signal through human AMHRII^40^. In these experimental conditions, AMH concentration in the medium ranged from 5 to 10 pM in fresh medium to about 10 to 15 pM after 5 days of culture (Fig. S2).

### Anti-AMH B10 antibody development and production

Three anti-AMH human scFv antibodies were selected by phage display from the human scFv phage display library Husc I^17,18^ after sequential panning using the Ala453-Arg560 AMH C-terminal mature peptide (cTER) domain (R&D). Antibodies were first expressed in the murine IgG2a format. The B10 antibody was selected for further experiments because it displayed the best binding to full-length LR-AMH^13^, as determined by ELISA.

For B10 antibody production, HEK293T cells were grown in 150 mm^2^ dishes up to 70% confluence. A 1:1 mixture of 30 µg of plasmid encoding B10 and 240 g of the transfection agent polyethylenimine (Polyscience) was kept at room temperature for 10 min, and then added to the cells for 6 h. Then, the transfection medium was replaced by DMEM without FBS. Five days later, supernatant was collected and diluted (1:1) with 40 mM sodium phosphate buffer, pH 8, filtered through a 0.22 µm filter and purified on a 1 ml protein A column for 24 h. Antibodies were eluted at acidic pH (glycine pH 3), and immediately stabilized with Tris buffer, pH 9.0. Centricon filters with a cut-off of 50 kDa were used to concentrate the antibody in PBS; 200 ml of cell culture provided about 1 mg of purified antibody.

### ELISA assay

ELISA was used to determine the EC_50_ of the B10 antibody. A 96-well high protein-binding capacity plate (Nunc MaxiSorp) was coated with polyclonal anti-AMH antibodies (Abcam ab84952) overnight. Then, the plate was washed 3 times and saturated with a PBS/0.01% Tween-20/2% BSA solution for 2 h. After each step, the plate was washed 3 times with PBS/0.01% Tween-20. After incubation with LR-AMH (25 nM) at 37 °C for 2 h, the B10 antibody (666–0 nM) was added at 37 °C for 1h30 followed by the secondary anti-Fc mouse peroxidase (HRP) antibody for 30 min, and finally the substrate enzyme (Thermofisher TMB). Absorbance was read at 450 nm after stopping the enzymatic reaction by the addition of sulfuric acid.

### Cell lines

The human COV434^41^ and KGN^42^ cell lines were kind gifts from Dr. PI Schrier (Department of Clinical Oncology, Leiden University Medical Center, Netherland) and Dr T Yanase (Kyushu University, Fukuoka, Japan), respectively. The human high grade serous ovarian cancer cell lines SKOV3 and NIH-OVCAR8 were from ATCC (ATCC HTB-77) and from the Division of Cancer Treatment and Diagnosis, NCI, Frederick, MD, USA, respectively. Cells were grown in DMEM/F-12 medium without red phenol containing 10% heat-inactivated FBS. COV434-AMHRII and SKOV3-AMHRII cells were supplemented with 0.33 mg/ml geneticin (InvivoGen, ant-gn-1). Cells were grown at 37 °C in a humidified atmosphere with 5% CO_2_, and medium was replaced twice per week. Cells were harvested with 0.5 mg/ml trypsin/0.2 mg/ml EDTA. All culture media and supplements were purchased from Life Technologies. Inc. (Gibco BRL). HEK293T cells, used for antibody production by the GenAc platform at IRCM, were grown in DMEM/F-12 with phenol red and 10% heat-inactivated FBS. The absence of *Mycoplasma* was tested every other week (MycoAlert LT07-318 & LT07-518, Lonza Walkersville, USA). The COV434-AMHRII and SKOV3-AMHRII cell lines were generated by transfection of the cDNA encoding full-length human AMHRII according to Kersual et al.^43^.

### Primary tumor cells from ascites

Ascites samples from two patients with ovarian cancer were obtained from the Institut du Cancer de Montpellier (ICM) according to the French laws and after their informed consent. All samples came from the ICM ovarian cancer clinical-biological database that had been approved by the independent “Sud Méditerranée III” ethics committee (study reference: 2016.09.06). All experimental protocols using these human samples were approved by the ICM CORT committee (COmité de Recherche Translationnelle) and were then carried out in accordance with the French guidelines and regulations for Human samples. These patients never received chemotherapy and were awaiting surgery. Freshly obtained ascites samples were aliquoted in 50 ml conical centrifuge tubes and spun at 1300 rpm for 5 min. Cell pellets were re-suspended in ammonium-chloride-potassium buffer (150 nM NH_4_Cl, 10 nM KHCO_3_, and 0.1 nM Na_2_EDTA) to lyse red blood cells on ice for 5 min. The process was repeated until lysis was complete. Then, cell pellets were plated on 150 mm cell culture dishes with 20 ml DMEM/F-12 with GlutaMAX (Gibco) and 10% FBS. The same day, 100,000 cells were harvested to assess AMHRII expression by FACS. Cells were then plated in DMEM/F-12/10% FBS for 30 min to rapidly eliminate adherent fibroblasts^44^. Non-adherent cells were transferred to new dishes with DMEM/F-12 and 10% FBS. Low-passage cells were used for experiments or frozen in liquid nitrogen.

### siRNA transfection

siRNA sequences were designed with the Rosetta algorithm by Sigma-Aldrich and a pool of three siRNAs was used. Cells were plated in 24-well plates to 60–80% confluence. Transfection was performed in medium with 1% FBS using Lipofectamine RNAiMax Transfection Reagent diluted in Opti-MEM Medium according to the provider (Thermofisher cat# 13778-150). siRNAs were diluted to 1 µg/ml (siRNAs against AMH, EHU121781) in Opti-MEM, and the siRNA-Lipofectamine (1:1) mixture was added to the cells for 6 h. Cells were washed and cultured in DMEM/F-12/1%FBS. Experiments with siRNA-transfected cells were performed at 24 h (COV434-AMHRII cells) or 48 h (SKOV3-AMHRII cells) post-transfection.

### Western blot analysis

Cells were washed with PBS and scraped immediately in RIPA lysis buffer (Santa Cruz) that included 200 mM PMSF solution, 100 mM sodium orthovanadate solution, and protease inhibitor cocktail. The protein concentration was determined using the BCA assay protein quantitation kit (Interchim). Cell extracts were heated at 95 °C for 5 min, separated (50 μg proteins/line) on 10% SDS-PAGE in reducing conditions (5% 2β-mercaptoethanol), and transferred to PVDF membranes (Biorad). Membranes were saturated in Tris-buffered saline, containing 0.1% Tween-20 and 5% non-fat dry milk, and probed with the relevant primary antibodies at room temperature for 1 h. After washing, peroxidase-conjugated IgG secondary antibodies were added (1/10,000) at room temperature for 1 h. After washing, antibody-antigen interactions were detected using a chemiluminescent substrate (Merck). To verify equal loading, immunoblots were also probed with an anti-GAPDH monoclonal antibody (Cell Signaling, Cat# 8884). Membrane exposition was performed using the G:BOX iChemi (Syngene) and did not exceeded 5 min. Image acquisition was performed using the GeneSys and its processing using ImageJ software.

### AMH pathway analysis

Cells were cultured in DMEM/F-12/1% FBS medium overnight, and then incubated with LR-AMH (0–25 nM) at 37 °C for 6 h. Western blotting was performed using anti-phosphorylated SMAD 1/5 (Cell Signaling, Cat# 9516), anti-phosphorylated AKT (Cell Signaling, Cat# 9271), anti-cleaved caspase 3 (Cell Signaling, Cat# 9661), anti-cleaved PARP (Cell Signaling, Cat# 9546), anti-GAPDH (1:1.000; Cell Signaling), anti-ALK2 (Abcam, Cat# ab60158), and anti-ALK3 (Abcam, Cat# ab38560) primary antibodies at 4 °C overnight, followed by anti-rabbit and anti-goat IgG HRP secondary antibodies (1:10.000; Sigma) at room temperature for 1 h.

### Cell viability assay

For cell viability/proliferation testing, the CellTiter 96 AQueous One Solution Cell Proliferation Assay system (Promega) was used according to the manufacturer’s instructions. Five thousand cells were plated in each well of a 96-well plate and cultured in 50 µl DMEM/F-12/1% FBS medium overnight. Cells were then incubated with LR-AMH (0-25 nM) or the anti-AMH B10 antibody (0–50 µg/ml) for 3 days. Then, 10 μl of CellTiter 96 AQueous One Solution reagent was added per well, and plates were incubated in humidified 5% CO_2_ atmosphere until the positive control wells became brown (from 1 to 2 h, depending on the cell line). Then, absorbance was measured at 490 nm using a PHERASTAR microplate reader. Three replicate wells were used for each condition.

### Clonogenic survival

Cells were plated in 24-well plates (50 cells/well) in DMEM/F-12/1% FBS medium overnight. LR-AMH (70 nM) was then added for 11 days. For COV434-AMHRII cells, which grow as clearly individualized clones, colonies were fixed in methanol/acetic acid solution (3:1) at 4 °C for 20 min, stained with 10% Giemsa, and counted. For SKOV3-AMHRII cells, the number of clones was estimated from the confluence area, determined using the Celigo Imaging System after cell staining with Hoechst 33342 trihydrochloride (Invitrogen H1399, 0.25 µg/ml for 15 min).

### Apoptosis induction assay

Apoptosis initiation was measured with the Caspase-Glos-3/7 assay (Promega, G8090). Cells were plated in white 96-well plates and incubated with LR-AMH (0–25 nM) for 6 h. Upon addition of the proluminescent caspase-3/7 DEVD-aminoluciferin substrate, caspase-3/7 cleavage of this substrate releases aminoluciferin that is consumed by luciferase to produce a luminescent signal, proportional to the caspase-3/7 activity. The luminescent signal was quantified with a PHERASTAR microplate reader 30 min after substrate addition.

### In vivo studies using ovarian cancer cell xenografts

All animal experiments were performed in compliance with the guidelines of the French government and Inserm regulations for experimental animal studies (agreement D34-172-27). All experimental protocols were approved by the CCEA-36 (Comité d’éthique en expérimentation animale Languedoc Roussillon) and were carried out in compliance with the ARRIVE guidelines. For all the in vivo experiments, 7.106 human COV434-MISRII cells 43 in BD Matrigel (ratio 1:1) in a volume of 150 µl were subcutaneously grafted on the right flank of female athymic nude Hsd mice (6–8 week-old) (ENVIGO, France), at day 0 (D0). Mice were randomized when tumor volume reached 60-150mm^3^, at D12-D13 (n = 5–7 mice/group). Treatments were all administered by intraperitoneal injection twice a week for 4 weeks. The anti-AMH MAb B10 (IgG2a format, produced in HEK296T cells) and the anti-AMHRII MAb 12G4 (chimeric IgG1 format, produced in CHO cells) were injected at 10 mg/kg. The untreated group received saline solution (vehicle). Tumor dimensions were measured with a caliper once per week, and tumor volumes were calculated using the formula: D_1_ × D_2_ × D_3_/2. Results were also expressed with an adapted Kaplan–Meier survival curve, using the time needed for a tumor to reach the volume of 1500 mm^3^. The median survival was defined as the time when 50% of mice had a tumor of 1500 mm^3^.

### Statistical analysis

Statistical analyses concerning differences in caspase-3/7 activity and cell viability/proliferation were performed with the Prism software and the non-parametric Kruskal–Wallis test except for Fig. 1a where the control group was only compared to the 0.8 nM AMH treated group. In this particular case, a Student’s t test was used as well as in Figs. 1d, 2b and 3b that involved only 2 groups (siAMH *vs* siMock and non treated *vs* B10 70 nM).

A linear mixed regression model was used to determine the relationship between tumor growth and the number of days post-graft. The fixed part of the model included variables corresponding to the number of days post-graft and the different groups. Interaction terms were built into the model. Random intercept and random slope were included to take into account the time effect. The coefficients of the model were estimated by maximum likelihood and considered significant at the 0.05 level^43^. Survival rates were estimated from the xenograft date until the date when the tumor reached the volume of 1500 mm^3^ using the Kaplan–Meier method. Median survival was presented with 95% confidence intervals. Survival curves were compared using the log-rank test^43^. Statistical analyses were carried out using the STATA 16.0 software (StataCorp, College Station, TX).

## Supplementary Information

Supplementary Information.
