# Supplementary material for: Anti-Müllerian hormone (AMH) autocrine signaling promotes survival and proliferation of ovarian cancer cells
Source: Sci Rep. 2021 Jan 26;11:2231. doi: 10.1038/s41598-021-81819-y (PMC7838181; doi:10.1038/s41598-021-81819-y)
Supplement: Supplementary file 1 — Supplementary Information. [file 41598_2021_81819_MOESM1_ESM.pdf]

Supplemental information

Antibody characterization, original Western blot and Drawings

Anti-Müllerian hormone (AMH) autocrine signaling promotes survival and proliferation of ovarian cancer cells

Maëva Chauvin[1,*], Véronique Garambois[1], Pierre-Emmanuel Colombo[1,2], Myriam Chentouf[1], Laurent Gros[1], Jean-Paul Brouillet[1,3], Bruno Robert[1], Marta Jarlier[1], Karen Dumas[4], Pierre Martineau[1], Isabelle Navarro-Teulon[1], David Pépin[5,6], Thierry Chardès[1], André Pèlegrin[1,✉]

[1]IRCM, Institut de Recherche en Cancérologie de Montpellier, Montpellier, F-34298, France; INSERM, U1194, Montpellier, F-34298, France; Université Montpellier, Montpellier, F-34298, France; Institut régional du Cancer de Montpellier, ICM, Montpellier, F-34298, France;

[2]Institut régional du Cancer de Montpellier, ICM, Montpellier, F-34298, France;

[3]Département de Biochimie et Biologie Moléculaire, CHU de Nîmes, Nîmes, France;

[4]SurgiMAb, 10 Parc Club du Millénaire, 1025 Avenue Henri Becquerel, 34000 Montpellier, France

[5]Department of Surgery, Harvard Medical School, Boston, MA, USA

[6]Pediatric Surgical Research Laboratories, Massachusetts General Hospital, Boston, MA, USA

✉Corresponding author at: Institut de Recherche en Cancérologie de Montpellier, Campus Val d'Aurelle, 34298 Montpellier Cedex, France; Tel: +33.4.67.61.30.32; FAX: +33.4.67.61.37.87; Email address: André Pèlegrin, andre.pelegrin@inserm.fr

*Present address: Pediatric Surgical Research Laboratories, Massachusetts General Hospital, Boston, MA, USA

S1 : B10 characterization

a

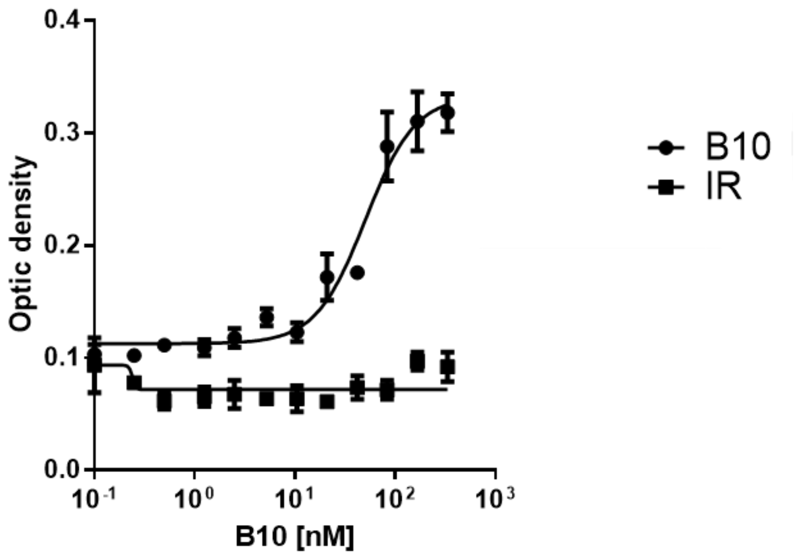

b

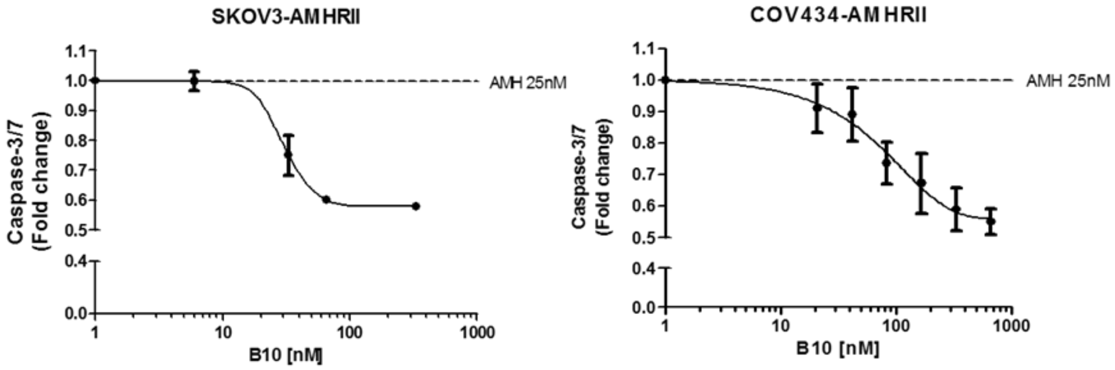

Figure S1. Characterization of the B10 anti-AMH monoclonal antibody. (A) For ELISA assays, plates were coated with an anti-AMH polyclonal antibody (Abcam Cat# ab84952) to capture AMH, and B10 was added at different concentrations to determine its EC_{50} (50.4±1.2 nM). IR, irrelevant antibody 13R4. (B) B10 inhibits apoptosis induced by incubation with 25 nM LR-AMH in COV434-AMHRII and SKO3-AMHRII cells.

S2 : AMH concentration in cell culture supernatants

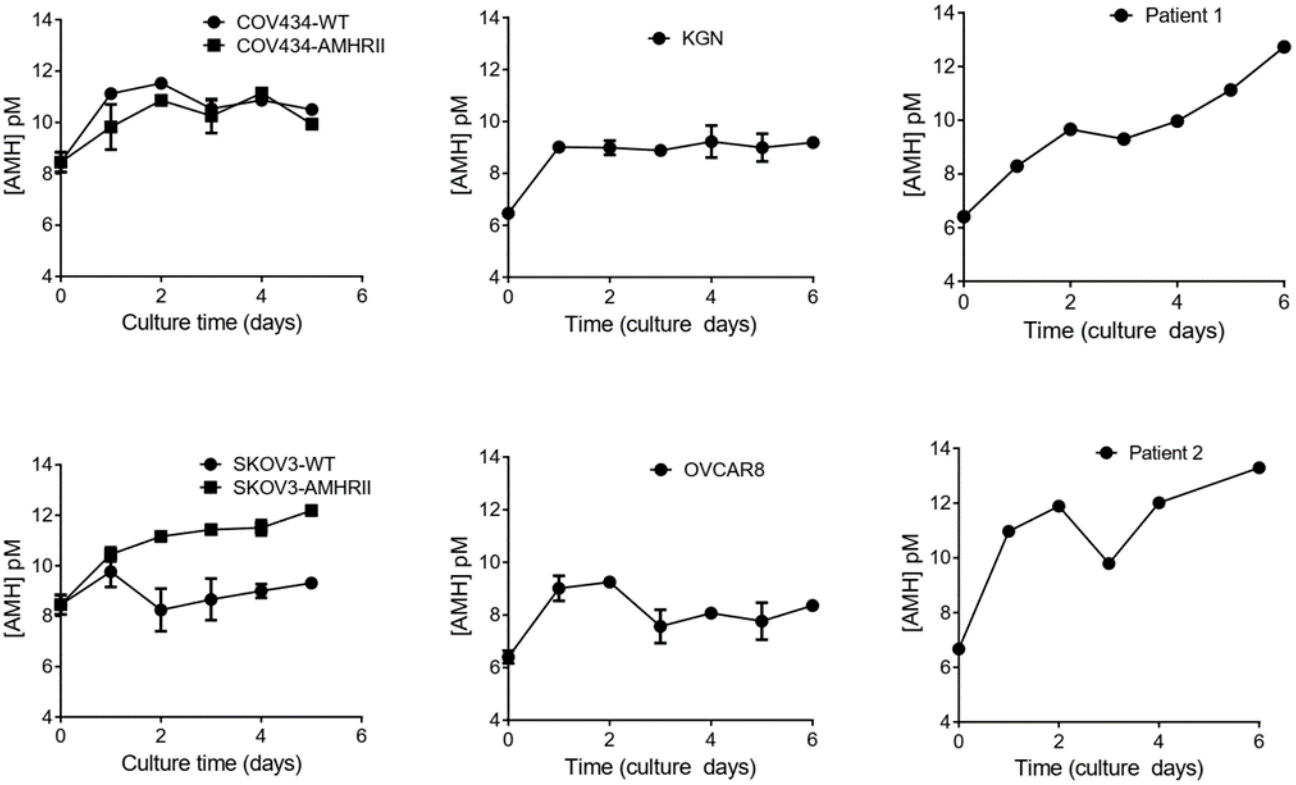

Figure 2. AMH concentration in basal cell cultures. All experiments involving LR-AMH were performed in culture medium containing 1% FBS in which AMH concentration ranged from 6 to 9 pM in fresh medium to about 10 to 15 pM after 5 days of cell culture. For all cell lines, graphs represent two independent experiments (mean±range)

S3 : Original Western Blot

Figure 1_panel b

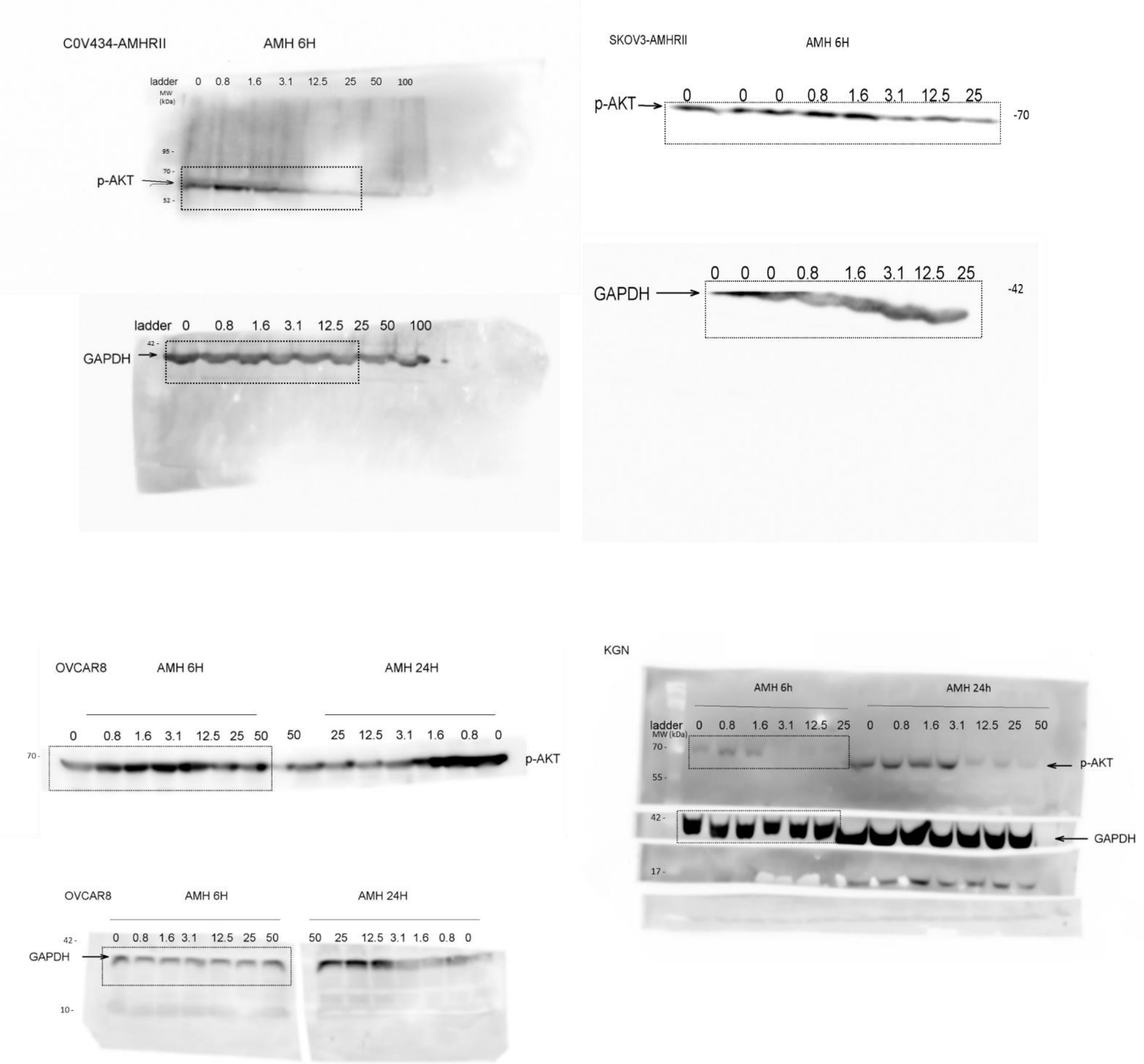

Figure 1_Panel c

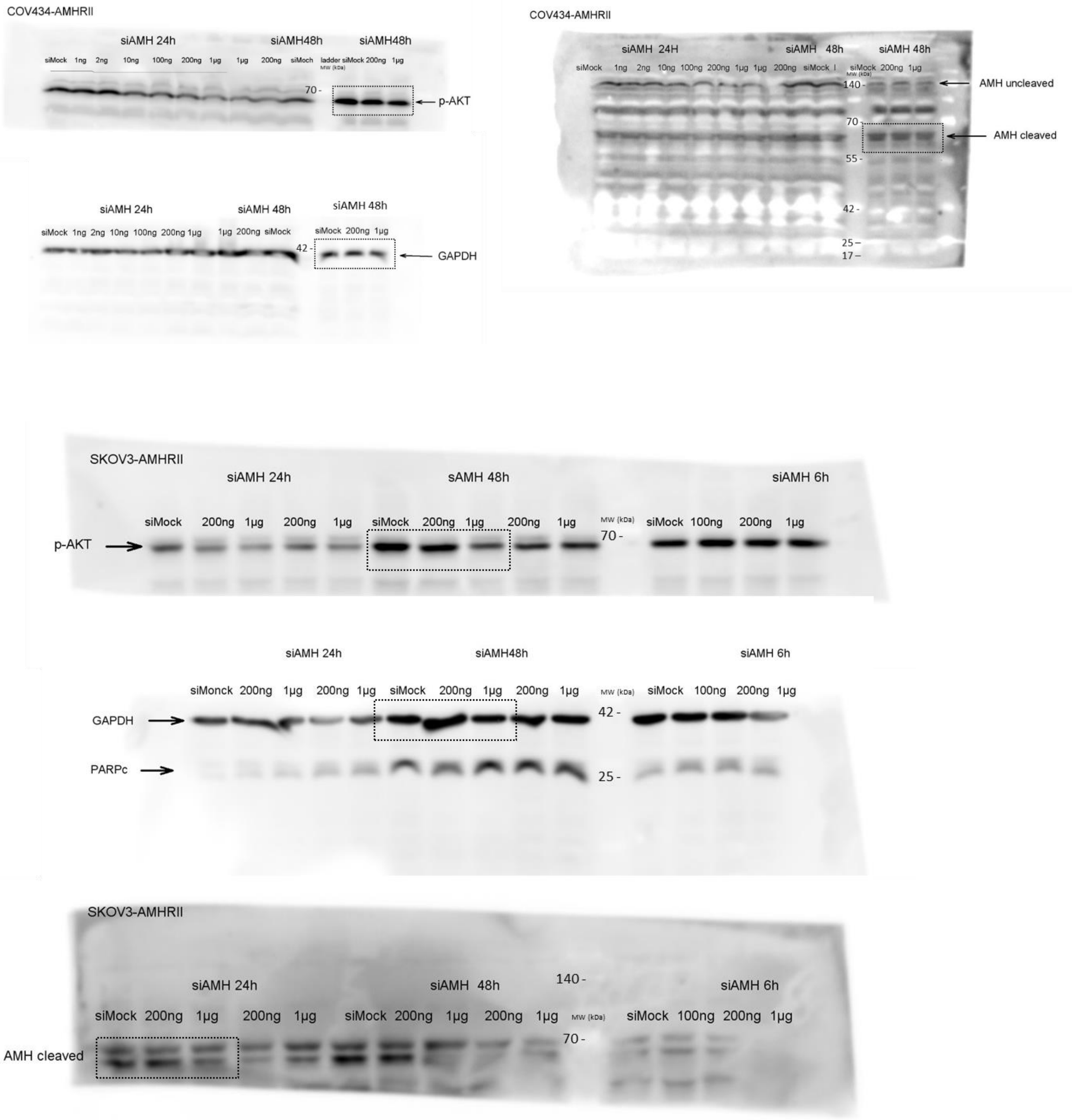

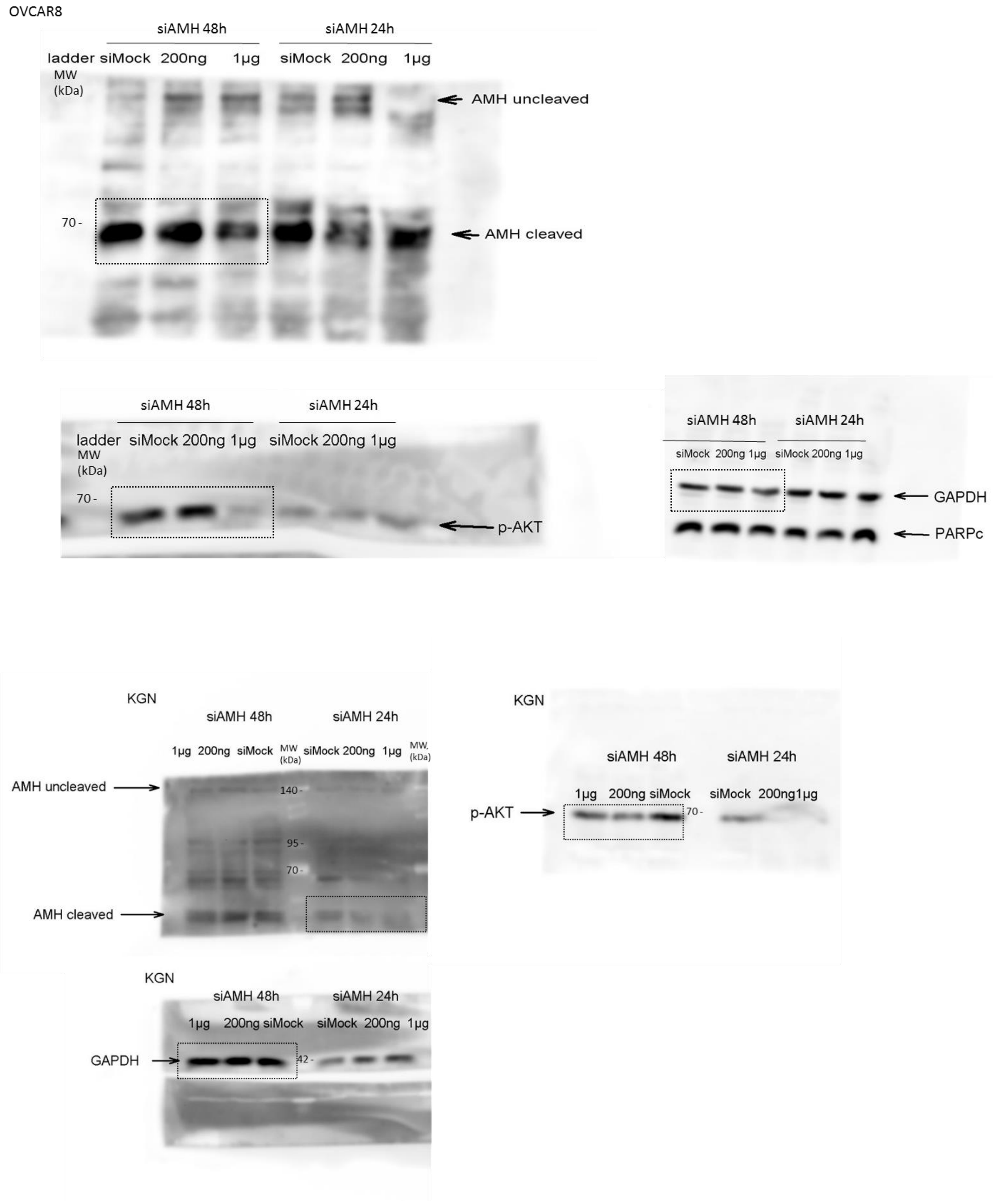
OVCAR8
siAMH 48h
siAMH 24h
ladder siMock 200ng 1µg siMock 200ng 1µg
MW (kDa)
70 -
AMH uncleaved
AMH cleaved
siAMH 48h
siAMH 24h
ladder siMock 200ng 1µg siMock 200ng 1µg
MW (kDa)
70 -
p-AKT
siAMH 48h siAMH 24h
siMock 200ng 1µg siMock 200ng 1µg
GAPDH
PARPc
KGN
siAMH 48h siAMH 24h
1µg 200ng siMock MW (kDa) siMock 200ng 1µg MW (kDa)
AMH uncleaved
140 -
95 -
70 -
AMH cleaved
KGN
siAMH 48h siAMH 24h
1µg 200ng siMock siMock 200ng1µg
p-AKT
70 -
KGN
siAMH 48h siAMH 24h
1µg 200ng siMock siMock 200ng 1µg
GAPDH
42 -

Figure 2_Panel c

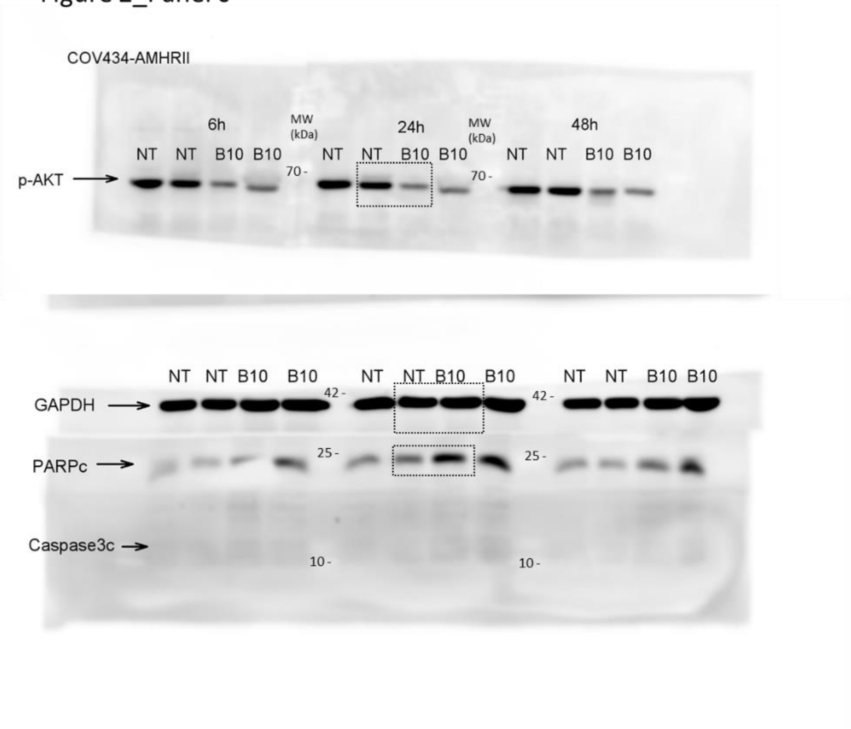

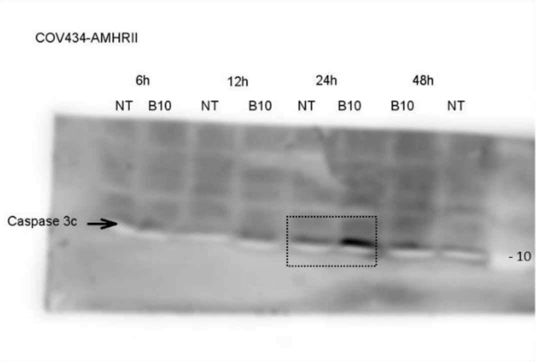

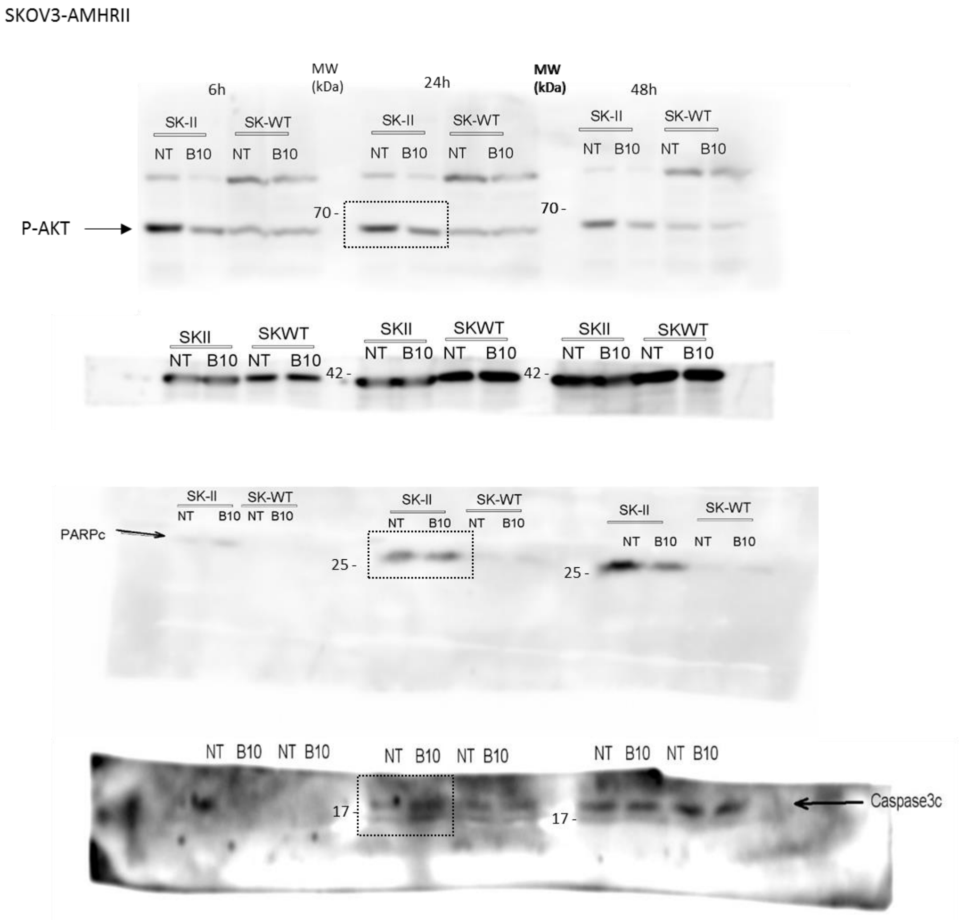

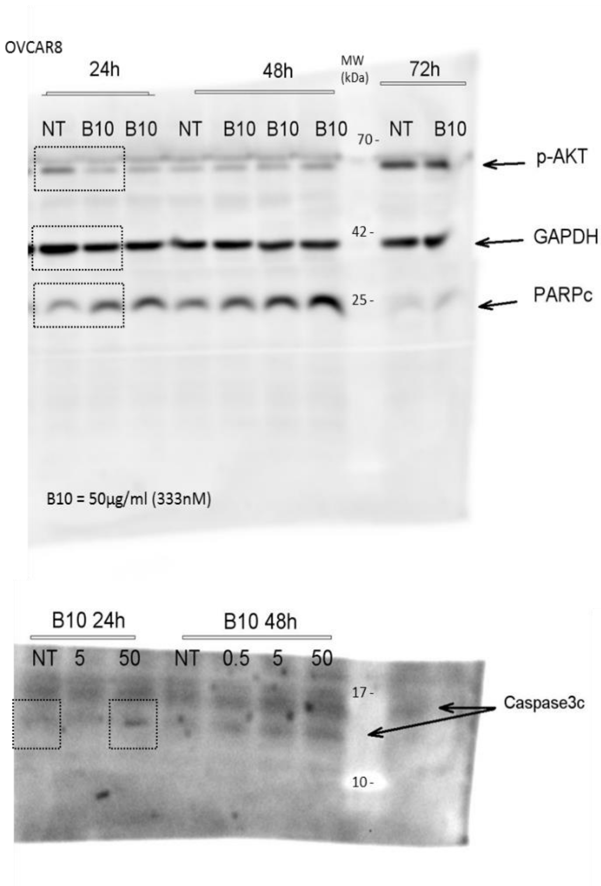
OVCAR8
24h
48h
MW (kDa)
72h
NT B10 B10 NT B10 B10 B10 NT B10
70 -
p-AKT
42 -
GAPDH
25 -
PARPc
B10 = 50µg/ml (333nM)
B10 24h
B10 48h
NT 5 50 NT 0.5 5 50
17 -
10 -
Caspase3c

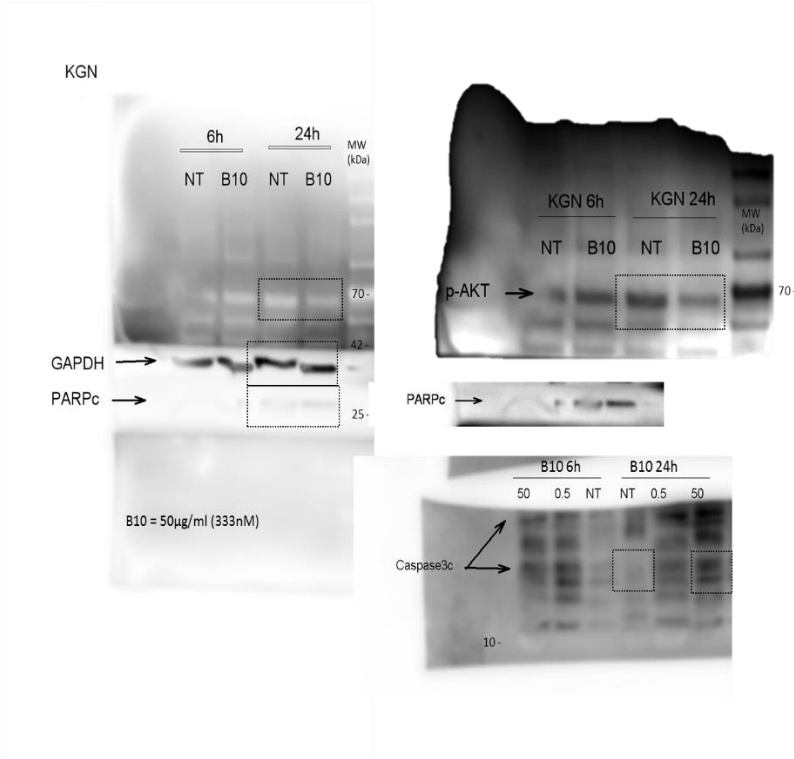
KGN
6h
24h
MW (kDa)
NT B10 NT B10
70 -
42 -
25 -
GAPDH
PARPc
B10 = 50µg/ml (333nM)
KGN 6h
KGN 24h
NT B10 NT B10
p-AKT
70 -
PARPc
B10 6h
B10 24h
50 0.5 NT NT 0.5 50
Caspase3c
10 -
